# Novel Mechanisms of Exercise-Induced Cardioprotective Factors in Myocardial Infarction

**DOI:** 10.3389/fphys.2020.00199

**Published:** 2020-03-10

**Authors:** Yuan Guo, Jingyuan Chen, Haihua Qiu

**Affiliations:** ^1^Department of Cardiovascular Medicine, The Affiliated Zhuzhou Hospital Xiangya Medical College, Central South University, Zhuzhou, China; ^2^Department of Cardiovascular Medicine, The Second Xiangya Hospital, Central South University, Changsha, China

**Keywords:** exercise, cardioprotective factors, exosomes, extracellular vesicles, myocardial infarction

## Abstract

Exercise training has been reported to ameliorate heart dysfunction in both humans and animals after myocardial infarction (MI). Exercise-induced cardioprotective factors have been implicated in mediating cardiac repair under pathological conditions. These protective factors secreted by or enriched in the heart could exert cardioprotective functions in an autocrine or paracrine manner. Extracellular vesicles, especially exosomes, contain key molecules and play an essential role in cell-to-cell communication via delivery of various factors, which may be a novel target to study the mechanism of exercise-induced benefits, besides traditional signaling pathways. This review is designed to demonstrate the function and underlying protective mechanism of exercise-induced cardioprotective factors in MI, with an aim to offer more potential therapeutic targets for MI.

## Introduction

Myocardial infarction (MI) is a serious result of cardiovascular disease and the leading cause of mortality and morbidity (Andersson et al., 2018). Approaches to inhibit the pathological process of MI are essential for its treatment and prognosis. To date, well-documented evidences have proved that appropriate exercise training can alleviate MI, which reduces mortality and improves the net clinical benefit in patients with MI (Anderson et al., 2016; Lear et al., 2017; Moholdt et al., 2018). Therefore, exercise training has been widely recommended as a therapeutic strategy for MI.

Various factors, including multiple polypeptides, nucleic acids, and similar substances are induced during exercise, which, in part, exert protective biological effects against several diseases (Whitham et al., 2018). Parts of cardioprotective factors are largely secreted by or enriched in the heart, which facilitate direct communication between the myocardium and other organs, and induce repair of cardiac injury under pathologic conditions (Shimano et al., 2012). Recently, it has been reported that the level of some cardioprotective factors is significantly increased during exercise training (Sanchis-Gomar et al., 2016; Whitham et al., 2018). Discovery and characterization of exercise-induced cardioprotective factors are of great interest because they may lead to a better understanding of the alterations in cell-to-cell communication in MI and help identify new therapeutic targets.

However, the secretory mechanisms of exercise-induced cardioprotective factors and their network regulation in MI are complex. Extracellular vesicles (EVs) released by cells are composed of a lipid bilayer enclosing soluble cytosolic material and nuclear components, which could act in an autocrine or paracrine manner to play a role in intercellular communication (Kowal et al., 2014). EVs divide into apoptotic bodies, microvesicles, and exosomes (EXs) depending on their size (Kowal et al., 2014). EVs, especially the most widely studied EXs, can be induced by exercise to facilitate the exchange of peptides, non-coding RNAs (ncRNAs), mRNA, DNA, and metabolites between cells and tissues, and are considered to play a key role in intercellular communication under physiological and pathological conditions, such as MI (Kowal et al., 2014; Davidson et al., 2017).

**FIGURE 1:**
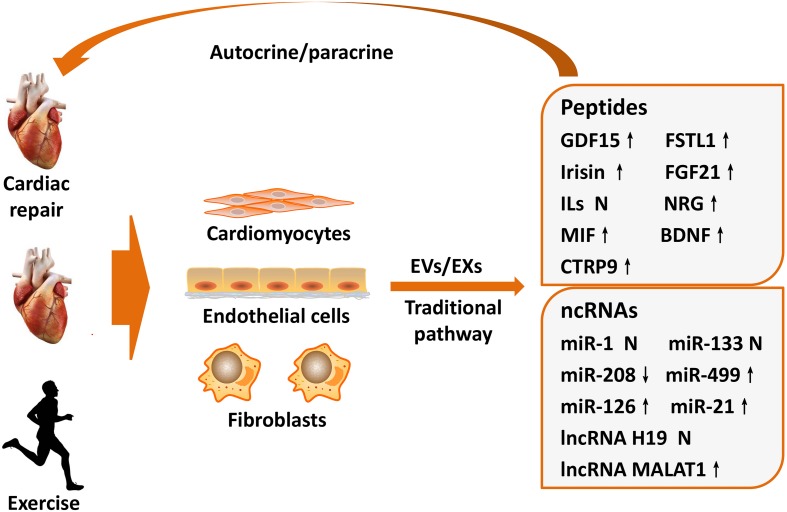
Exercise-induced cardioprotective factors on cardiac repair after MI. The heart is an endocrine organ that produces and releases cardioprotective factors during exercise training. Cardioprotective factors mainly derived from cardiomyocytes, endothelial cells, and cardiac fibroblasts, which not only secreted by traditional signaling pathways but also delivered via EVs/EXs that changed components during exercise. Subsequently, these factors exert important regulatory functions for cardiac repair after MI in autocrine or paracrine manners, which may partially explain exercise-induced beneficial mechanism of MI. BDNF, brain-derived neurotrophic factor; CTRP9, C1q/TNF-related protein 9; EVs, extracellular vesicles; EXs, exosomes; FSTL1, follistatin-like 1; FGF21, fibroblast growth factor 21; GDF15, growth differentiation factor 15; ILs, interleukins; lncRNA, long non-coding RNA; MI, myocardial infarction; MIF, migration inhibitory factor; miR, microRNA; MALAT1, metastasis associated lung adenocarcinoma transcript 1; NRG, neuregulin; ncRNAs, non-coding RNAs. ↑ means upregulate, ↓ means downregulate, N means not clarified or controversial.

Intriguingly, circulatory EVs/EXs content varies in an intensity-dependent manner in response to exercise training (Bei et al., 2017). The heart is the main endocrine organ affected during exercise, and some cardiac-derived protective factors can be induced by exercise and carried by EVs/EXs to ameliorate the pathological processes of MI (Ogawa and de Bold, 2014; Sanchis-Gomar et al., 2016). Thus, this review summarizes the protective role and underlying mechanism of cardiac-derived protective factors that can be induced by exercise in MI, with an aim to demonstrate their potential as therapeutic targets (Figure 1).

## Exercise-Induced Peptides and MI

Certain cardiac-derived peptides exert multiple biological functions under pathophysiological states. It has been reported that exercise can induce the secretion of various cardioprotective factors, which exert protective effects in MI (Safdar et al., 2016) (Table 1).

**TABLE 1:** Exercise-induced peptides mediate MI.

Names	Secreted cells	Functions	Main modulation mechanisms	References
GDF15	Cardiomyocytes	Inhibit inflammation	Repress PMN recruitment by directly inhibiting chemokine signaling and integrin activation	Kempf et al., 2011
FSTL1	Fibroblasts, cardiomyocytes	Inhibit inflammation and cardiac fibrosis, promote angiogenesis	Attenuate the migratory and proliferative capabilities of cardiac fibroblasts and expression of extracellular matrix proteins	Maruyama et al., 2016
Irisin	Cardiomyocytes	Reduce apoptosis; induce cardiac regeneration	Suppress the opening of mitochondrial permeability transition and protect mitochondria function; promote the function of cardiac progenitor cells	Wang H. et al., 2017; Zhao et al., 2019
FGF21	Cardiomyocytes, cardiac endothelial cells	Attenuate ventricular remodeling and myocyte apoptosis, increase capillary density	Decrease pro-inflammatory cytokines levels in an adiponectin-dependent manner and decrease miR-145-mediated autophagy	Joki et al., 2015; Hu S. et al., 2018
IL-33	Cardiac fibroblasts	Inhibit apoptosis and inflammation, reduce cardiac fibrosis	Suppress macrophage infiltration and production of inflammatory cytokines, inhibit NF-κB and p38 MAPK pathways, induce M2 macrophage polarization	Yin et al., 2014; Li et al., 2019
NRG	Cardiac endothelial cells	Attenuate apoptosis	Attenuate endoplasmic reticulum stress by activating PI3K/AKT pathway	Fang et al., 2017
MIF	Cardiomyocytes, cardiac fibroblasts	Promote cardiomyocytes survival and regulate inflammation	Promoted CSCs survival, proliferation and endothelial differentiation, activate PI3K/AKT/mTOR and AMPK pathways	Cui et al., 2016
BDNF	Endothelial cells, myocardial cells	Promote angiogenesis, inhibit inflammation and ventricle remodeling	Targeting its functional receptor, tyrosine receptor kinase B	Wang et al., 2018
CTRP9	Cardiac endothelial cells	Attenuate cardiomyocyte death	Activation of ERK/MMP-9 and ERK/Nrf2 signaling, upregulation of anti-oxidative proteins	Yan et al., 2017

*BDNF, brain-derived neurotrophic factor; CTRP9, C1q/tumor necrosis factor-related protein-9; CSCs, cardiac stem cells; ERK, extracellular signal-regulated kinase; FSTL1, follistatin-like 1; FGF21, fibroblast growth factor 21; GDF15, growth differentiation factor 15; IL, interleukin; MI, myocardial infarction; MIF, migration inhibitory factor; miR, microRNA; MAPK, mitogen-activated protein kinase; mTOR, mammalian target of rapamycin; MMP, matrix metalloproteinases; NRG, neuregulin; NF-κB, nuclear factor-κB; PMN, polymorphonuclear leukocytes; PI3K/AKT, phosphatidylinositol-3-kinase/protein kinase B.*

### Growth Differentiation Factor 15 (GDF15) and Follistatin-Like1 (FSTL1)

Growth differentiation factor 15 (GDF15), also known as macrophage inhibitory cytokine 1, is a polypeptide molecule that could be synthesized and secreted by cardiomyocytes (Shimano et al., 2012; Emmerson et al., 2017). GDF15 expression is induced under various pathophysiological states and significantly increased during MI onset (Khan et al., 2009). It has also been found that higher levels of GDF-15 in patients with acute coronary syndrome are associated with raised risks of spontaneous MI, as well as cardiovascular and total mortality (Hagström et al., 2016). Thus, GDF15 could serve as both a biomarker and predictor for the prognosis of MI.

Moreover, GDF15 expression can be induced by exercise. A recent study reported that plasma GDF15 levels in healthy male individuals significantly increased from 215 pg/mL at rest to 295 pg/mL at the end of the exercise bout, and further increased to about 350 pg/mL at the end of recovery (Kleinert et al., 2018). More interestingly, GDF15 from femoral artery and femoral vein has been, respectively, tested before, during, and after exercise and found no difference. This finding suggests that exercise-induced GDF15 may not be secreted by the skeletal muscle (Kleinert et al., 2018), but possibly be derived from other organs, especially the cardiac muscle.

Growth differentiation factor 15 has been found to play important roles in regulating the pathophysiological process of MI via its vital anti-inflammatory function (Kempf et al., 2011). Excessive inflammatory cell recruitment to the infarcted area after MI aggravates myocardial remodeling and leads to fatal complications. Kempf et al. (2011) reported that GDF15-deficient mice showed a significant increase in the recruitment of polymorphonuclear leukocytes (PMNs) to the infarcted myocardium and an increase in the incidence of cardiac rupture. Conversely, infusion of recombinant GDF15 in MI mice repressed PMN recruitment by directly inhibiting chemokine signaling and integrin activation, indicating that GDF15 protects against fatal cardiac rupture after MI though controlling inflammatory cell recruitment. Rainer et al. (2014) further showed that suppression of cardiomyocyte-specific transforming growth factor (TGF)-β promoted GDF15 expression, which inhibited neutrophil integrin activation and heart remodeling after infarction (Rainer et al., 2014). Although GDF15 is increasing after MI and its higher level can predict a worse prognosis, it may be a promising exercise-induced therapeutic target for MI.

FSTL1, a member of the follistatin family, is abundantly expressed in the skeletal and cardiac muscle, and has been shown to mediate multiple aspects of MI (Wei et al., 2015; Shen et al., 2019). Maruyama et al. (2016) found that FSTL1 expression significantly increased after MI, and inhibiting its expression increased mortality due to cardiac rupture in the acute phase of MI. Moreover, FSTL1 deficiency-induced cardiac rupture was mainly associated with attenuation of the migratory and proliferative capabilities of cardiac fibroblasts and reduction of extracellular matrix proteins mediated via the extracellular signal-regulated kinase (ERK)1/2 pathway (Maruyama et al., 2016). Shen et al. (2019) showed that FSTL1 expression declined dramatically in hypoxia-induced mesenchymal stem cells (MSCs), while overexpression of FSTL1 significantly prolonged MSC retention after implantation in the ischemic myocardium, thereby preserving heart function after MI by limiting scar formation, reducing inflammatory response, and enhancing neovascularization. These findings suggest that FSTL1 might serve as a key therapeutic target for MI.

Exercise training has been shown to induce FSTL1 expression (Xi et al., 2016; Kon et al., 2019a). In healthy individuals, 60 min of cycling dramatically increased the serum FSTL1 level by 22%, with a concentration increase from 16.9 ng/mL (pre-exercise) to 20.1 ng/mL (immediately after exercise), which further increased to 21.9 ng/mL at 30 min after completing the exercise [23]. More interestingly, exercise training further increases ischemia-induced FSTL1 expression. A previous study showed that FSTL1 expression in MI mice was 1.96-fold higher than that in control mice, and it further increased by 4.04-fold after intermittent aerobic exercise (Xi et al., 2016). In addition, exercise-induced FSTL1 has been shown to improve cardiac remodeling and prognosis after MI via promoting angiogenesis and reducing cardiac fibrosis (Xi et al., 2016).

Interestingly, it was previously detected by a luciferase-based reporter gene assay that FSTL1 as an upstream regulator of GDF-15. It has been found that treatment with FSTL1 activated GDF15 production in cultured cardiomyocytes. More importantly, transgenic production of FSTL1 stimulated GDF15 production in the murine heart, whereas cardiomyocyte selective deletion of FSTL1 decreased production of GDF15 in cardiomyocytes, suggests that these proteins function as components of an interactive network (Widera et al., 2012). Moreover, the circulating concentration of FSTL1 has been shown to be dependently related to GDF15 and act as a biomarker to predict cardiovascular mortality in patients with acute coronary syndrome (Widera et al., 2012). Overall, the co-expression of FSTL1 and GDF15 provides an explanation for the mechanism of exercise-induced cardioprotection in MI, at least in part.

### Irisin and Fibroblast Growth Factor 21 (FGF21)

Irisin, a new type of muscle factor highly expressed in the myocardium, has been considered as a novel exercise-induced cardioprotective factors (Tsuchiya et al., 2015; Wang H. et al., 2017). Irisin levels have been shown to be significantly induced by various exercise types. Plasma irisin levels were increased by 65% in mice after 3 weeks of free wheel running exercise, while circulating irisin levels were increased by twofold in healthy adult humans after 10 weeks of supervised endurance exercise training compared to the non-exercising group (Bostrom et al., 2012). More interestingly, the effects of different exercise duration and models on irisin levels are distinct. Irisin levels were shown to increase significantly after acute strenuous exercise and a 30 min bout of intensive exercise in children and young adults, but remained unchanged after 6 weeks of chronic exercise training (Loffler et al., 2015). Endurance exercise significantly increased irisin levels, which began to decrease after 2 h of exercise (Nygaard et al., 2015).

Recently, irisin has been found to protect against the pathologic process of MI via its anti-apoptotic, pro-angiogenetic, and cardiac regenerative functions (Wang H. et al., 2017; Liao et al., 2019; Zhao et al., 2019). Wang H. et al., 2017 showed that irisin treatment induced remarkable improvements in ventricular functional recovery and reduction of infarct size in the Langendorff perfused ischemia/reperfusion (I/R) injury heart of mice via suppressing the opening of mitochondrial permeability transition pore, which results in mitochondrial swelling, and protecting mitochondrial function to reduce cardiomyocyte apoptosis. Liao et al. (2019) reported that treatment with irisin for 2 weeks significantly reduced infarct size and fibrosis in MI mice, significantly increased angiogenesis in the ischemic area, and decreased cardiomyocyte apoptosis via activating the ERK signaling pathway. Besides, it has been recently reported that irisin can induce cardiac regeneration and functional improvement of MI mice via promoting the function of cardiac progenitor cells (CPCs) (Zhao et al., 2019). Thus, irisin is a key exercise-induced cardioprotective factor exerting multiple functions in MI.

FGF21 has been found to ameliorate the pathological progression of MI (Joki et al., 2015; Hu S. et al., 2018; Tang et al., 2018). It has been shown that injection of MI mice with recombinant interleukin (IL)-22 in the 1st week after acute MI effectively prevents left ventricular (LV) dysfunction and attenuates ventricular remodeling via markedly increasing FGF21 expression in a signal transducer and activator of transcription (STAT)3-dependent manner, indicating that FGF21 might be a promising therapeutic target for MI treatment (Tang et al., 2018). Another recent study showed that increased FGF21 expression can ameliorate cardiac remodeling in MI mice via increasing capillary density around the infarct area and reducing cardiomyocyte apoptosis together with decreasing pro-inflammatory cytokine level in an adiponectin-dependent manner (Joki et al., 2015). Besides, FGF21 has been reported to protect against I/R-injury via decreasing miR-145-mediated autophagy (Hu S. et al., 2018).

Circulating FGF21 levels have been shown to significantly increase with exercise training (Geng et al., 2019). In healthy subjects, the level of FGF21 increases from 276.8 to 460.8 ng/L after 2 weeks of physical activity (Cuevas-Ramos et al., 2012). Another random cross-sectional study showed that 5 weeks of endurance exercise significantly increased circulating FGF21 levels in the elderly, and decreased their liver fat content (Taniguchi et al., 2016). It has also been speculated that exercise may induce cardiac-specific FGF21 expression via the sirtuin1 (Sirt1)/peroxisome proliferator-activated receptor gamma coactivator-1α (PGC-1α) signaling pathway (Guo et al., 2016). Thus, FGF21 is considered as an important exercise-induced cardioprotective factor for MI treatment.

It has been widely considered that irisin is widely expressed in cardiac and skeletal muscle, while FGF21 is also abundantly exist in the liver, adipose tissue, skeletal and cardiac muscle to mediate lipid metabolism. A previous study identified that exercise-induced irisin secretion could interact with FGF21 to exert biological functions, suggesting that they may regulate muscle–adipose crosstalk during exercise (Lee et al., 2014). Thus, their regulatory network may be a way to explain muscle–adipose or organ-organ crosstalk during exercise training, and thereby indicating further mechanisms of exercise-induced benefits in MI.

### IL Family

Several members of the IL family have been identified as cardioprotective factors, some of which are induced by exercise. IL-33, a cytokine belonging to the IL-1 family, is abundantly expressed in the heart, and attenuates inflammatory response and acts as the specific ligand for soluble ST2 (Shimano et al., 2012; Chen et al., 2018). IL-33 is mainly secreted by cardiac fibroblasts, exerting its function in a paracrine manner to exchange information with cardiomyocytes, and thereby playing an important role in the pathophysiological process of MI (Chen et al., 2018).

It has been reported that IL-33/ST2 pathway activation results in inhibition of apoptosis and inflammation, reduction of cardiac fibrosis, and improvement of cardiac function (Chen et al., 2018). Yin et al. (2014) found that IL-33 effectively suppressed macrophage infiltration and production of inflammatory cytokines in the myocardium after MI, and injection of recombinant IL-33 in MI mice reduced infarct size, attenuated cardiac remodeling, and improved cardiac function by inhibiting nuclear factor-κB (NF-κB) and p38 mitogen-activated protein kinase (MAPK) signaling pathways. Besides, Li et al. (2019) showed that IL-33 reduced infarct area and prevented the progression of fibrosis by inducing M2 macrophage polarization in MI mice model via activating the Janus kinase (JAK)/STAT pathway. Thus, IL-33 exerts anti-inflammatory effects in MI.

Various factors, including exercise training, have be shown to stimulate IL-33 expression. Further, it has been demonstrated that long-term medium-intensity exercise not only decreases the expression of pro-inflammatory factors, like Toll-like receptor 4 (TLR4), NF-κB, and IL-18, but also significantly increases the level of the anti-inflammatory factor IL-33 in patients with diabetes mellitus (Liu et al., 2015). Hence, IL-33 is considered as an exercise-induced cardioprotective factor that exerts important protective functions in MI.

Besides, IL-6 and IL-1β have also been identified as cardioprotective factors, as they aggravate MI. Distinctly, exercise training has been shown to reduce their expression, which may be a potential approach to ameliorate MI (Pedersen, 2017). Although not all IL family members are widely expressed in the heart and induced by exercise, they exert vital functions to regulate the course of MI. Several members of this family have been identified as exercise-induced cardioprotective factors, and may serve as promising therapeutic targets for MI.

### Other Exercise-Induced Polypeptides

Neuregulin (NRG) is expressed in human cardiac endothelial cells and acts as an endothelial cell-derived molecule that exerts cardioprotective effects (Hedhli et al., 2011). NRG has been found to protect against cardiomyocyte apoptosis induced by hypoxia-reoxygenation, and in an *in vivo* study it was shown to reduce infarct size and cardiomyocyte apoptosis after myocardial I/R-injury (Hedhli et al., 2011). Furthermore, the cardioprotective effect of NRG was shown to be mediated via attenuation of endoplasmic reticulum stress and cardiomyocyte apoptosis by activating the phosphatidylinositol-3-kinase/protein kinase B (PI3K/AKT) signaling pathway under I/R states (Fang et al., 2017). More interestingly, exercise can upregulate NGR and its ligand expression to promote cardiac repair, indicating that NRG is an exercise-induced cardioprotective factor and serves as a promising therapeutic target for MI (Cai et al., 2016).

Migration inhibitory factor (MIF) is a macrophage factor that regulates inflammation and immunity, and is secreted by cardiomyocytes and cardiac fibroblasts to promote cardiomyocyte survival and regulate inflammation after MI (Voss et al., 2019). Besides, it has been reported that MIF promotes cardiac stem cell survival, proliferation, and endothelial differentiation by targeting its receptor CD74 via activation of the PI3K/AKT/mammalian target of rapamycin (mTOR) and adenosine monophosphate-activated protein kinase (AMPK) signaling pathways. This finding suggests a potential therapeutic role of MIF in the treatment of MI (Cui et al., 2016). Additionally, MIF expression can also be induced by exercise, and it has been identified as an exercise-induced cardioprotective factor that protects against MI (Chang et al., 2019).

Brain-derived neurotrophic factor (BDNF), widely expressed in various non-neural tissues, such as vascular endothelial cells and myocardial cells, plays a protective role in MI via targeting its functional receptor, tyrosine receptor kinase B (Zhang et al., 2019). Recent studies have found that BDNF can promote angiogenesis, inhibit inflammatory response, and attenuate cardiac remodeling, thereby improving cardiac function and prognosis after MI (Wang et al., 2018). Well-documented evidences have shown that BDNF expression is significantly induced by exercise training, and thereby it can be considered as a beneficial exercise-induced cardioprotective factor (Wang et al., 2018; Zhang et al., 2019).

C1q/TNF-related protein-9 (CTRP9) is a novel cardioprotective factor primarily secreted by the adipose tissue and cardiac endothelial cells (Zhao et al., 2018). CTRP9 has been shown to enhance adipose-derived mesenchymal stem cell (ADSC) proliferation and survival after implantation to MI mice. Further, CTRP9 stimulates ADSC migration and attenuates cardiomyocyte cell death after MI via binding with N-cadherin, activation of ERK/MMP-9 and ERK/Nrf2 signaling, and upregulation/secretion of anti-oxidative proteins (Yan et al., 2017). Besides, CTRP9 expression is also induced by exercise, and a single bout of high intensity interval training is sufficient to stimulate CTRP9 secretion in healthy men (Kon et al., 2019b). Thus, CTRP9 has been identified as a novel exercise-induced cardioprotective factor exerting potential therapeutic effects in MI.

## Exercise-Regulated ncRnas and MI

Non-coding RNAs, which do not code for proteins but functionally regulate protein expression, have been identified as critical regulators of cell function and important candidates that protect against MI (Guo et al., 2017). MicroRNAs (miRNAs, miRs) are a class of short-chain ncRNAs that have been reported to regulate MI via negatively modulating their target genes (Chistiakov et al., 2016). Cardiac-derived miRNAs induced by exercise may explain the exercise-induced beneficial effects on MI (Gomes et al., 2014) (Table 2).

**TABLE 2:** Cardiac-enriched ncRNAs regulate MI.

ncRNAs	Regulated by exercise	Functions	Main modulation mechanisms	References
miR-1	Controversial	Inhibit apoptosis, promote cardiomyogenic differentiation	Activate PTEN/AKT pathway and improve transplanted MSC survival rate	Glass and Singla, 2011; Huang et al., 2013
miR-133	Controversial	Attenuate cardiomyocyte apoptosis and cardiac fibrosis, promote angiogenesis and cardiomyocytes proliferation	Inhibit pro-apoptotic genes DAPK2 expression, improve transplanted MSC survival rate	Izarra et al., 2014; Li et al., 2015; Chen et al., 2017
miR-208	Downregulate	Aggravate cardiac fibrosis, promote apoptosis	Induce apoptosis through regulation of target gene Ets1, directly proportional to β-MHC and cardiac collagen capacity	Bian et al., 2015
miR-499	Upregulate	Inhibit cell apoptosis	Inhibit mitochondrial apoptosis pathway, inhibit pro-apoptotic gene Dyrk2, reduce the dephosphorylation of Drp1 and the accumulation of Drp1 in mitochondria	Wang et al., 2011, 2014
miR-126	Upregulate	Promote angiogenesis, maintain vascular wall integrity	Inhibit its target gene Spred1	Guo et al., 2018
miR-21	Upregulate	Inhibit fibrosis, inflammation, and apoptosis	Inhibit its target gene Jagged1, inhibit KBTBD7, p38 and NF-κB pathway and TNF-α induced apoptosis	Wang Z.H. et al., 2017; Yang et al., 2018; Zhou X.L. et al., 2018
lncRNA H19	Normalized H19 gene methylation	Reduce necrosis and cardiac remodeling, enhance angiogenesis, activate autophagy	Target to miR-103/107, miR-139, and miR-675-5p, respectively	Wang et al., 2015; Gong et al., 2017; Huang P. et al., 2019
lncRNA MALAT1	Upregulate	Promote angiogenesis, cells proliferation and autophagy, inhibit apoptosis, increase cardiac fibrosis	Sponge miR-558, miR-145, and miR-200a-3p, respectively	Guo et al., 2019; Huang S. et al., 2019; Sun and Zhang, 2019

*DAPK2, death associated protein kinase 2; Drp1, dynamin-related protein-1; KBTBD7, kelch repeat and BTB (POZ) domain containing 7; lncRNA, long non-coding RNA; MI, myocardial infarction; miR, microRNA; MALAT1, metastasis associated lung adenocarcinoma transcript 1; MSC, mesenchymal stem cell; NF-κB, nuclear factor-κB; PTEN, phosphatase and tensin homolog deleted on chromosome 10; TNF-α, tumor necrosis factor-α.*

### miR-1 and miR-133

miR-1 and miR-133 are highly expressed in cardiomyocytes and play an important role in regulating myocardial autonomy, conduction, contraction, and myocyte differentiation and proliferation (Chistiakov et al., 2016). Previous studies have reported that miR-1 and miR-133 expression is regulated by exercise. It was shown that miR-1 and miR-133 expression significantly increased in marathon athletes after exercise (Gomes et al., 2014). However, another study demonstrated that miR-1 and miR-133 expression was significantly downregulated after exercise (Pietrangelo et al., 2015). These paradoxical results may be attributed to individual variations and differences in exercise duration. More interestingly, both miR-1 and miR-133 have been shown to exert important regulatory functions in MI (Duan et al., 2018; Yu et al., 2019).

While the regulatory role of miR-1 in MI remains controversial, studies suggest that transplantation of stem cells with miR-1 overexpression exerts important protective effects after MI. It has been reported that transplantation of embryonic stem cells with miR-1 overexpression can significantly reduce apoptosis and improve cardiac function via phosphatase and tensin homolog deleted on chromosome 10 (PTEN)/AKT pathway activation (Glass and Singla, 2011). Additionally, transplantation of MSCs overexpressing miR-1 into the infarcted myocardium of mice was shown to improve cell survival rate, promote cardiomyogenic differentiation, and improve cardiac function, indicating that miR-1 has a protective effect on MI (Huang et al., 2013). However, the direct function of miR-1 in MI requires further investigation.

miR-133 plays a cardioprotective function in MI. Li et al. (2015) found that overexpression of exogenous miR-133 significantly attenuated cardiomyocyte apoptosis, both *in vitro* and *in vivo*, in a hypoxia-reoxygenation injury model via inhibition of death associated protein kinase 2 (DAPK2). Izarra et al. (2014) further reported that miR-133 can attenuate myocardiocyte apoptosis through inhibiting the expression of pro-apoptotic genes, including caspase-9, apoptotic protease activating factor, DAPK2, Bcl2-like 11, and Bcl-2-modifying factor. In an MI rat model, it was reported that overexpression of miR-133 significantly promotes angiogenesis and cardiomyocyte proliferation, and inhibits cardiac hypertrophy and fibrosis, thereby improving cardiac function (Izarra et al., 2014). Another study showed that transplantation of miR-133-overexpressing MSCs in ischemic area in MI rat model markedly improved cardiac function (Chen et al., 2017). Therefore, overexpression of miR-133 may be an important therapeutic strategy for MI treatment.

### miR-208 and miR-499

The cardiac-enriched miRNAs, miR-208 and miR-499, are mainly involved in modulation of differentiation and development of cardiac fibroblasts and cardiomyocytes, and play a vital role in maintaining normal cardiac function (Chistiakov et al., 2016). It has been shown that the expression of miR-208 is significantly downregulated, while that of miR-499 is markedly upregulated after exercise training, which may partly explain the benefits of exercise (Baggish et al., 2014; Soci et al., 2016).

miR-208 promotes cardiomyocyte apoptosis and cardiac remodeling after MI. Yan et al. (2016) found that inhibition of miR-208 expression in neonatal rat cardiomyocytes under hypoxia condition could relieve cardiomyocyte injury. Bian et al. (2015) showed that overexpression of miR-208 markedly aggravated I/R-induced myocardial injury in rats and promoted hypoxia-induced cardiomyocyte apoptosis *in vitro*, while knockdown of miR-208 suppressed cardiomyocyte apoptosis. Further, they found that miR-208 induced apoptosis via its target gene Ets1. The expression of miR-208 is directly correlated with β-MHC levels and cardiac collagen capacity. Therefore, inhibiting miR-208 expression may be a potential therapeutic approach for MI.

miR-499 has important regulatory effects on the pathological process of MI. Wang et al. (2014) found that miR-499 protected cardiomyocytes against H_2_O_2_-induced apoptosis and overexpression of miR-499 in rat cardiomyocytes increased cell survival rate by inhibiting the mitochondrial apoptosis pathway and pro-apoptotic gene expression. The pro-apoptotic gene Dyrk2 promoted apoptosis by increasing the level of p53 phosphorylation, whereas miR-499 significantly inhibited Dyrk2 expression, preventing the transfer of activated p53 to the mitochondria and inhibiting apoptosis. miR-499 has also been shown to suppress calcineurin-mediated dephosphorylation of dynamin-related protein-1 (Drp1) to inhibit cardiomyocyte apoptosis, thereby reducing Drp1 accumulation in the mitochondria and mitochondrial fission (Wang et al., 2011). Furthermore, Shi et al. (2019) found that miR-499 reduced H9C2 cell injury by inhibiting the expression of Sox6, and suppressed apoptosis by increasing Bcl-2 and decreasing Bax and caspase-3 expression under hypoxia-reoxygenation condition. Therefore, miR-499 can target multiple genes to protect against apoptosis after MI.

### Other Cardiac-Derived miRNAs

miR-126, mainly expressed in endothelial cells, significantly promotes angiogenesis around the infarct area after MI (Jiang et al., 2014). The expression of cardiac-enriched miR-126 is significantly changed after MI, exerting pro-angiogenetic effects to maintain vascular wall integrity by negatively regulating its target gene Spred1 (Guo et al., 2018). Exercise can increase miR-126 expression. It has been reported that miR-126 expression was upregulated by 2.1- and 4.6-fold in healthy subjects after a maximal symptom-limited exercise test and 4 h of cycling, respectively (Uhlemann et al., 2014). Thus, miR-126 is an important exercise-induced factor that protects against MI mainly by promoting angiogenesis.

miR-21, a miRNA transcribed by RNA polymerase II, is widely expressed in endothelial cells and exerts protective functions after MI. A previous study demonstrated that miR-21 exerts anti-fibrotic effects via promoting cardiac fibroblast-to-myofibroblast transformation by regulating its target gene Jagged1 (Zhou X.L. et al., 2018). Another study showed that miR-21 attenuated inflammation, cardiac dysfunction, and maladaptive remodeling post-MI through targeting kelch repeat and BTB (POZ) domain containing 7 and inhibiting p38 and NF-κB pathway activation (Yang et al., 2018). Intriguingly, a recent study showed that the serum level of miR-21 was upregulated in elderly patients with acute MI, and that miR-21 suppressed TNF-α-induced apoptosis in human cardiomyocytes via stimulating the activation of JNK/p38/caspase-3 signaling pathway (Wang Z.H. et al., 2017). Additionally, miR-21 expression has been shown to be regulated by exercise, indicating a novel mechanism underlying exercise-induced benefits in MI (Wahl et al., 2016).

### Long ncRNAs (lncRNAs)

lncRNAs are transcripts longer than 200 nucleotides that regulate various biological processes by interacting with multiple transcription factors (Wang et al., 2015). Recently, it has been identified that lncRNAs play a crucial regulatory function in MI. The lncRNAs H19 and metastasis associated lung adenocarcinoma transcript 1 (MALAT1) have been widely studied, and their levels are shown to be regulated by exercise. Exercise-induced lncRNA H19 expression has been shown to involve in epigenetic modifications (Xu et al., 2017). lncRNA MALAT1 expression can be induced by swimming via inhibition of apoptotic functions, thereby protecting hippocampal neurons against ischemic diseases (Shang et al., 2018).

The lncRNA H19 has been reported to play multiple roles after MI. Wang et al. (2015) reported that lncRNA H19 directly binds to miR-103/107 and reduces its expression, which promotes Fas-associated protein with death domain expression, and participates in H_2_O_2_-induced necrosis in H9C2 cells. This finding revealed that lncRNA H19 prevents cardiomyocyte necrosis in MI. Furthermore, lncRNA H19 was shown to alleviate myocardial cell injury through reduction of miR-139 expression and further upregulation of its target gene Sox8 via PI3K/AKT/mTOR and MAPK pathway activation (Gong et al., 2017). Additionally, lncRNA H19 has been shown to enhance angiogenesis, prevent cardiomyocyte apoptosis, and improve cardiac function after MI via targeting miR-675-5p (Huang P. et al., 2019). Besides, overexpression of lncRNA H19 in mice was shown to reduce infarct size and improve cardiac function via activating autophagy (Zhou M. et al., 2018). These findings demonstrate that lncRNA H19 exerts essential protective functions in MI.

lncRNA MALAT1 is highly expressed in vascular endothelial cells and has a complex function in MI (Li L. et al., 2018). It was initially reported that genetic deletion or pharmacological inhibition of MALAT1 reduced vascular growth *in vivo*, suggesting that MALAT1 promoted angiogenesis in ischemic diseases (Michalik et al., 2014). Besides, it has been shown that MALAT1 protects against cardiomyocyte apoptosis after MI by sponging miR-558, thereby inducing Unc-51-like autophagy-activating kinase 1-dependent protective autophagy (Guo et al., 2019). However, a recent study reported that MALAT1 promoted cardiac fibrosis and deteriorated cardiac function post-MI by increasing TGF-β1 activity and inhibiting miR-145 expression (Huang S. et al., 2019). Additionally, MALAT1 has been shown to increase cell apoptosis in MI via acting as a competing endogenous RNA to sponge miR-200a-3p (Sun and Zhang, 2019). Thus, lncRNA MALAT1 exerts critical regulatory functions in various biological processes during MI, including angiogenesis, cell proliferation, apoptosis, and cardiac fibrosis. Nevertheless, whether it plays a protective or detrimental role in MI requires further investigation.

## Novel Secretory Mechanisms of Exercise-Induced Cardioprotective Factors in MI

Recent researchers have identified EVs as an important cell-to-cell communication way (Doroudgar and Glembotski, 2011). EVs, secreted by cells in the form of vesicles containing various signaling molecules, play a role in cell-to-cell communication under pathophysiological conditions (Safdar et al., 2016). As previously described, EXs are one of the most widely studied EVs. Generally, EXs act in an endocrine, autocrine, or paracrine manner to exert their biological functions (Safdar et al., 2016).

The biogenesis and release of EXs is related to complex regulatory factors. As previously described, EXs biogenesis begins within the endosome system and is matured in an endosomal sorting complex required for transport (ESCRT)-dependent or independent manner (Colombo et al., 2014). Molecular regulators implicated in exosome release include multiple factors, especially Soluble NSF Attachment Protein Receptor (SNARE) and Rabs GTPases (Colombo et al., 2014). Thus, EXs carries not only secreted factors but also a cluster of scaffold proteins, which may be used to derive their source cells. It has been found that EXs originated from cardiomyocytes mainly express caveolin-3 and Troponin T, from cardiac fibroblast express CD90.2, and from endothelial cells express CD31 (Loyer et al., 2018).

At present, many kinds of cells have been shown to secrete EXs, including various stem cells, endothelial cells, cardiofibroblasts, and cardiomyocytes (Barile et al., 2017). EXs derived from different cells exhibit different functions. A recent study reported that cardiomyocytes release EVs under ischemic stress; and cardiomyocytes as the main source of bioactive EXs in coronary serum (Li H. et al., 2018). Interestingly, certain cells exert various biological functions by releasing EXs with different components, which varies under different culture conditions and stimuli (Gallet et al., 2017; Ribeiro-Rodrigues et al., 2017; Huang P. et al., 2019).

Previous studies have shown that EVs/EXs stimulated by ischemia exert important cardioprotective functions after MI via changing their contents (Gallet et al., 2017; Ribeiro-Rodrigues et al., 2017; Gao et al., 2018). Ribeiro-Rodrigues et al. (2017) found that EXs secreted by cardiomyocytes under ischemic conditions contained high levels of matrix metalloproteinases (MMP), miR-222, and miR-143, and stimulated the formation of new functional vessels following MI. Barile et al. (2014) reported that infarcted hearts injected with EVs from CPCs showed reduced cardiomyocyte apoptosis, enhanced angiogenesis, and improved LV ejection fraction compared with those injected with control medium, and this effect was induced by the changed levels of miR-210, miR-132, and miR-146a-3p in EVs. Li H. et al., 2018 also found that, compared to EXs from healthy controls, EXs from patients with myocardial ischemia enhanced endothelial cell proliferation, migration, and tube formation via downregulation of miR-939-5p, and thereby increased endothelial nitric oxide production, eventually promoting angiogenesis.

More interestingly, exercise can induce various organs to release EVs/EXs that are enriched in various peptides, ncRNAs, and other substances. The release of EXs was shown to significantly increase immediately after cycling exercise and then decline again within 90 min at rest, while release of EXs was moderate but appeared more sustained after treadmill running. Moreover, release of EXs into the circulation has been shown to be initiated in the aerobic phase, suggesting that it is independent of the metabolic changes during exercise (Fruhbeis et al., 2015). Further, in another study, after a 1 h bout of cycling exercise in healthy humans, an increase in the circulation of over 300 proteins was observed, and most of them were secreted by EXs, suggesting that exercise exerts systemic biological effects (Whitham et al., 2018). Thus, EXs may provide a new approach to explain the multiple protective mechanisms of exercise in MI.

Additionally, it has been found that exercise can induce cardiomyocytes to release EVs/EXs, which exert a cardioprotective function in MI. A recent study showed that the serum level of EVs was increased by about 1.85-fold in mice after 3 weeks of swimming (Bei et al., 2017). Furthermore, intramyocardial injection of EVs induced by exercise exerted additional anti-apoptotic effects on H_2_O_2_-treated H9C2 cardiomyocytes compared to exercise alone. The protective effects on acute ischemia in mice were mediated by the activation of the ERK1/2 and HSP27 pathways (Bei et al., 2017). Thus, exercise-induced EVs/EXs released by cardiomyocytes exert important protective functions in MI.

Cardioprotective factors carried by EXs have been found to regulate MI pathology (Hu M. et al., 2018). In an MI mice model, it was reported that the level of cardiac-enriched miRNAs, such as miR-1 and miR-499 was predominantly increased in circulating EXs, subsequently mediating functional crosstalk between the ischemic heart and bone marrow for repair of cardiac injury (Cheng et al., 2019). Recently, the exosomal lncRNA H19 derived from MSCs has been reported to mediate the cardioprotective effects in infarcted hearts via promoting endothelial cell function and angiogenesis (Huang P. et al., 2019). Besides, polypeptide molecules, like MIF and BNDF can also be delivered by EXs to exert biological functions (Suire et al., 2017; Amosse et al., 2018). Thus, EXs may serve as important vehicles to deliver exercise-induced cardioprotective factors and as mediators of intercellular communication in MI, which may be a possible mechanism underlying exercise-induced benefits.

In summary, exercise has beneficial effects on MI, but its mechanisms are complex and need to be fully elucidated. It has been found that exercise can induce the secretion of a variety of polypeptide molecules, ncRNAs, and other substances derived from the myocardium and other organs. Several recent studies have reported that EVs/EXs are enriched in a large number of cardioprotective factors, and their secretion is regulated by exercise. This review summarizes the effects of newly discovered exercise-induced cardiogenic peptides and ncRNAs on MI and their potential mechanisms, thus aiming to provide a new theoretical basis for the application of exercise training in the clinical treatment of MI.

## Author Contributions

YG drafted the manuscript. JC and HQ checked and revised the manuscript. All authors read and approved the final manuscript.

## Conflict of Interest

The authors declare that the research was conducted in the absence of any commercial or financial relationships that could be construed as a potential conflict of interest.
